# Time-Dependent Optothermal Performance Analysis of a Flexible RGB-W LED Light Engine

**DOI:** 10.3390/mi16091007

**Published:** 2025-08-31

**Authors:** Md Shafiqul Islam, Mehmet Arik

**Affiliations:** 1 Department of Mechanical Engineering, Auburn University, Auburn, AL 36849, USA; arik@auburn.edu; 2 EVATEG Center, Ozyegin University, Istanbul 34794, Türkiye

**Keywords:** light emitting diode (LED), luminous flux, luminous efficacy, junction temperature

## Abstract

The wide application of light emitting diodes (LEDs) in lighting systems has necessitated the inclusion of spectral tunability by using multi-color LED chips. Since the lighting requirement depends on the specific application, it is very important to have flexibility in terms of the driving conditions. While many applications use single or rather white color, some recent applications require multi-spectral lighting systems especially for agricultural or human-medical treatment applications. These systems are underexplored and pose specific challenges. In this paper, a mixture of red, green, blue, white (RGB-W) LED chips was used to develop a compact light engine specifically for agricultural applications. A computational study was performed to understand the optical distribution. Later, attention was turned into development of prototype light engines followed by experimental validation for both the thermal and optical characteristics. Each LED string was driven separately at different current levels enabling an option for obtaining an infinite number of colors for numerous applications. Each LED string on the developed light engine was driven at 300 mA, 500 mA, 700 mA, and 900 mA current levels, and the optical and thermal parameters were recorded simultaneously. A set of computational models and an experimental study were performed to understand the optical and thermal characteristics simultaneously.

## 1. Introduction

Solid-state lighting technology has experienced rapid advancements over the years and for that reason the extended adoption of LEDs has been possible in numerous lighting applications including indoor, architectural, agricultural, human-centric, etc. Indoor lighting has significant impact on energy saving and sustainability [1]. Progress in the field of LEDs has made a remarkable contribution towards energy-efficient and more sustainable indoor lighting solutions. The functionality of modern lighting has reached beyond its primary function of illumination e.g., visible light communication has also been explored via LEDs [2]. These dual roles of LEDs (i.e., illumination and data transmission) have the potential to revolutionize the lighting systems in modern architecture.

The advancement in the field of lighting technology has enabled numerous applications of LEDs beyond mere indoor and architectural lighting. Being the primary environmental factor for plant growth and development, lighting plays a crucial role in agriculture and due to increasing food demand across the world, cultivation is now not only dependent on sunlight. Due to the availability of a wide color spectrum and energy efficiency, LEDs are widely used as lighting sources in agriculture. This rich spectral distribution shows different distinctive effects on plant growth and development [3]. Hence, the next-generation agricultural lighting is continuously evolving around LED technologies. However, in order to enhance productivity, automation, and sustainability in indoor agriculture, it is necessary to integrate technologies from different domains such as control systems, sensing, and the internet of things (IoT) based automation, etc. [4]. The use of LEDs in plant lighting dates to the 1980s when a lighting system was developed for space shuttle and space stations with a view to mastering indoor farming [5]. Over the years, LED technology has advanced and using LEDs in indoor farming has become economically feasible because of their greater energy efficiency compared to other counterparts. Neo et al. [6] discussed and assessed spectral shaping and tuning and hybrid lighting technologies to generate high and consistent crop yields in controlled environment agriculture (CEA). They also discussed color conversion films in manipulating the light spectrum for plant growth. To obtain highly energy-efficient agricultural lighting, studies have been conducted to propose novel designs such as beam shaping by using a micro-lens diffuser [7].

While lighting is mostly explored in the context of indoor or architectural lighting, the area of human-centric lighting is still underexplored. Apart from visual effects, the non-visual effects of lighting on human physiology are also significant [8]. For instance, lighting plays a vital role in shaping human sleep quality, alertness, mode, and behavior. Hence, modern lighting research has expanded significantly and focused on the non-visual aspects in the context of human-centric lighting. Different human-centric designs and concepts have been developed and new metrices are also proposed for human-centric luminaires for creating a lighting design guide so that energy-efficient, sustainable, and human-centered lighting becomes feasible [9,10,11].

Since LED has firmly established its place as a semiconductor lighting source, mixed-color illumination is becoming popular and evolving around numerous applications. Hou et al. [12] discussed an RGB-based mixed color illumination system for microscopy and machine vision. Image quality was compared under both monochromatic light and mixed color illumination. RGB-W LEDs also contribute to developing new pixel architecture which can result in more energy efficiency, long lifespan, wider color gamut, and improved saturation levels in display [13,14]. In addition to that, RGB-W LED combinations have also been used for color controlling of a lighting system [15]. RGB-W enables color tunability with optimized color rendering and efficacy [16]. 

Several studies are to be found in the literature that examined the performance degradation of RGB-W LEDs over time due to several factors. Singh et al. [17] performed a study where they reported 30% lumen degradation after only 144 h of operation. They suggested RGB mixing can be a better solution for producing white light rather than using phosphor on blue LED. However, no reliability test was conducted in their study. Myland et al. [18] monitored LED degradation by using a multi-channel spectral sensor. Using the spectral sensor, even without detailed characterization of the sensor itself, allowed for an accurate monitoring of the true emission of LEDs, with a maximum radiometric error of 0.73%, a maximum colorimetric error of 0.0017 Δu′v′ and a maximum spectral nRMSE error of 0.0097 compared to a spectroradiometric measurement. Hamon et al. [19] also studied LED degradation from component to system level and predicted gradual output degradation as a function of test time. The time-dependent testing or investigations available in the literature are mostly focused on reliability assessment and lifetime prediction. However, the simultaneous optothermal performance evaluation with respect to time is very limited and especially rare for an application-specific LED luminaire. The variation of optical performance parameters such as luminous flux, luminous efficacy, radiant flux, and spectral flux etc. with time and junction temperature is significant and requires comprehensive analysis to understand the dynamic interplay between the thermal and optical behavior of a wide spectrum LED light engine. Hence, this paper presents a comprehensive time-dependent investigation of the optothermal behavioral interplay in an application-specific LED light engine.

In this study, an RGB-W LED light engine was developed, and the time-dependent nature of various optothermal performance parameters were analyzed. In the existing literature, the time-dependent analysis of optothermal parameters is very limited. Hence, this study provides important insights regarding the optothermal behavioral changes over the entire period of light engine operation. While most of the studies in the literature analyze the optical or thermal side separately, this study integrates both the thermal and optical sides to provide a deeper understanding on LED performance under realistic operating conditions. The comprehensive optothermal characterization of a custom-designed light engine will help to obtain application-specific performance goals which are not possible with off-the-shelf commercial luminaires.

## 2. Experimental Study

**Figure 1:**
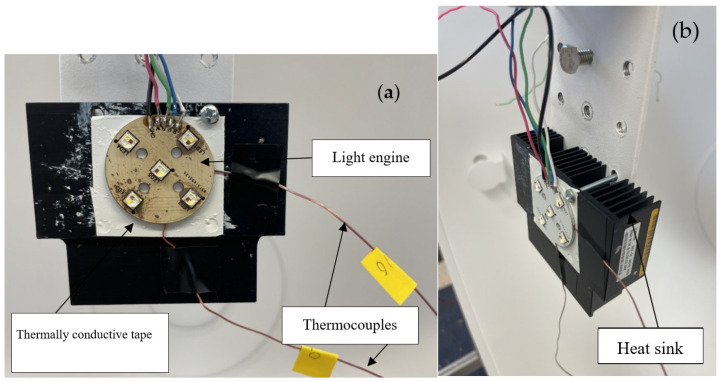
(**a**) Light engine components, (**b**) heat sink.

In this study, a mixture of different colors over a single light engine was investigated. The goal of the study was to develop a high-power LED light engine on a single printed circuit board (PCB) which can provide a wide range of a spectrum. Although there are some commercially available light engines containing multiple color LED chips, most of them are not capable of providing high lumen output. Hence, in a practical setting, multiple light engines are used to obtain the required luminous flux. This study focused on providing luminous flux as high as 1300 lm from a single compact LED light engine. Therefore, instead of using multiple light engines, this approach can be a sustainable solution for a high flux requirement, consuming less space and power. CREE X-Lamp XM-L Color Gen 2 High Density LEDs were used to develop the light engine. Based on the optical analysis, five LED chips were used to develop the light engine. Same color LEDs were connected in series so that the red, green, blue, and white LEDs could be driven separately. A Chroma DC power supply (Model: 62012P-600-8, Chroma ATE Inc., Taoyuan City, Taiwan) was used to drive the light engine at multiple current levels (300 mA, 500 mA, 700 mA, 900 mA). Figure 1 presents the light engine setup for conducting the experiments.

A heat sink was used under the PCB for driving the light engine at higher current levels. For the appropriate contact between the PCB and the heat sink, a thermally conductive adhesive tape (Ttape 1000 A, manufacturer: Laird [20]) was used which has a thickness of 0.05 mm and thermal conductivity of 0.70 W/m-K. For the optical analysis, the light engine was placed inside an integrating sphere (Illumia plus 40’’ base sphere, Labsphere, North Sutton, NH, USA), and a CDS600 USB2+U17543 spectrometer (Labsphere, North Sutton, NH, USA) was used for running real-time optical scans.

**Figure 2:**
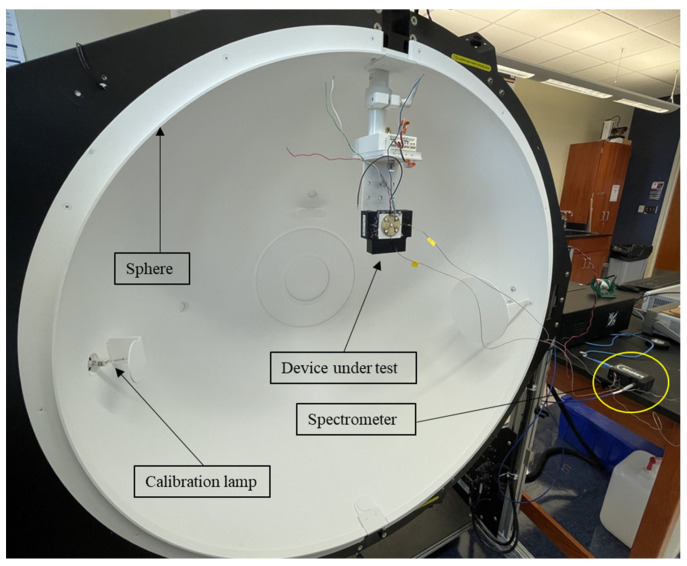
Integrating sphere setup for optical experiments.

Figure 2 presents the important components of the integrating sphere system. The CDS 600 spectrometer has a wavelength range of 200–850 nm with an integration time of 1 ms to 5 s. The spectral resolution is 2 nm with wavelength accuracy < 0.5 nm. The detector used in this spectrometer is a Sony ILX511 linear silicon CCD array with a range of 200–1100 nm. The highly reflective inner wall of the sphere is coated with barium sulfate which helps to reflect more than 95% of the incident light diffusely. Thus, the light scatters uniformly in all directions after several bounces inside the sphere.

**Figure 3:**
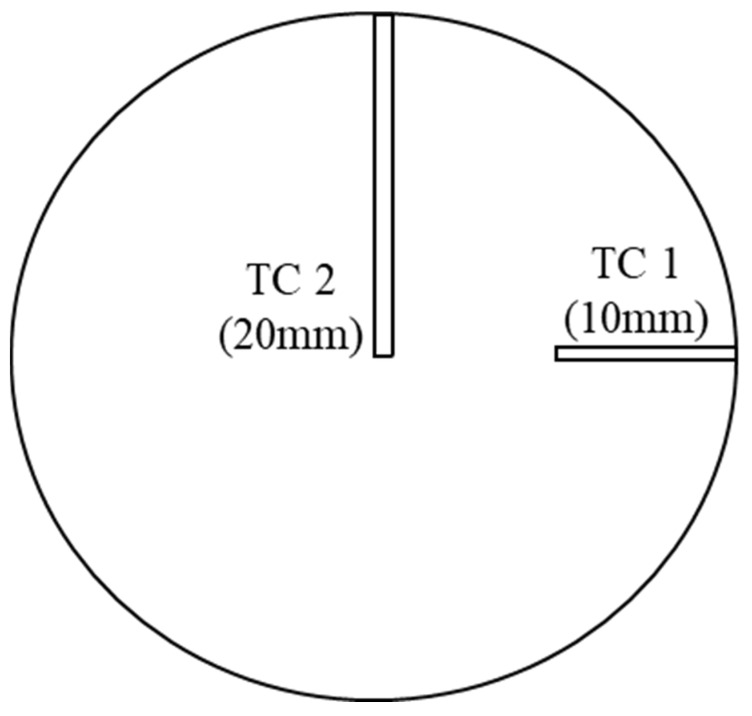
Thermocouple insertion at the back side of the PCB.

For analyzing the real-time thermal performance of the light engine corresponding to the optical performance, two T-type thermocouples (Omega TT-T-30-72, Omega Engineering, Norwalk, CT, USA) were inserted at the backside of the PCB. Two rectangular grooves with a depth of 0.70 mm were cut on the backside of the PCB in order to insert the thermocouple probe precisely. TC 1 (corresponds to temperature T_1_) was inserted 10 mm away from the periphery of the board while TC 2 (corresponds to temperature T_2_) was inserted 20 mm away from the periphery of the board, i.e., at the center of the PCB bottom. Figure 3 presents the details of the thermocouple insertion. This approach was explained in detail in our previous experiments [21,22].

While the integrated sphere software (Integral version 5.0) was used to run optical scans inside the integrating sphere, an Agilent data acquisition system (model: 34970A, Agilent Technologies, Santa Clara, CA, USA) captured the temperatures simultaneously. Each LED string (i.e., red, green, blue, and white) was driven for 30 min and data was recorded at 2 min intervals to observe the real-time optothermal performance of the light engine. 

After running each LED series for 30 min, the light engine was turned off and left idle for dissipating the heat and to reach ambient temperature. Data were collected from 5 scans per second and the average of those was used for the transient data.

**Figure 4:**
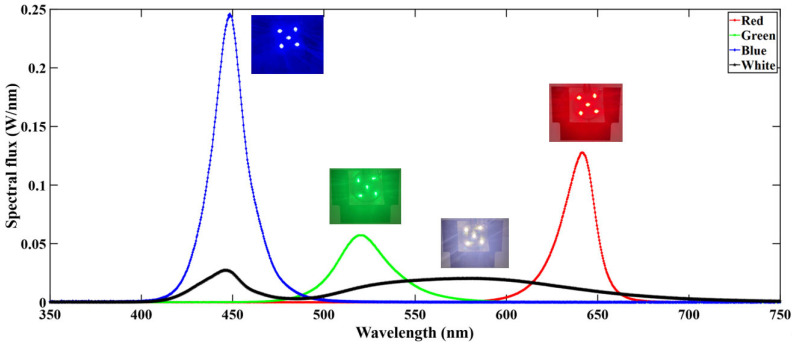
Spectral distribution of the light engine at 900 mA current after 30 min of operation.

Figure 4 represents a typical spectral distribution curve of the light engine. This curve was obtained at 900 mA current driving condition at room ambient and after operating for 30 min. The curve shows that the blue LED series provides the highest spectral flux followed by red, green, and white. The light engine reached steady state after operating for 25–30 min.

## 3. Computational Models

**Figure 5:**
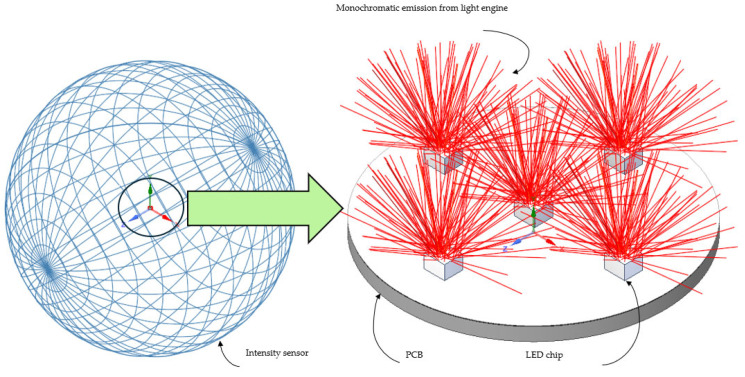
Simplified optical model along with intensity sensor.

**Figure 6:**
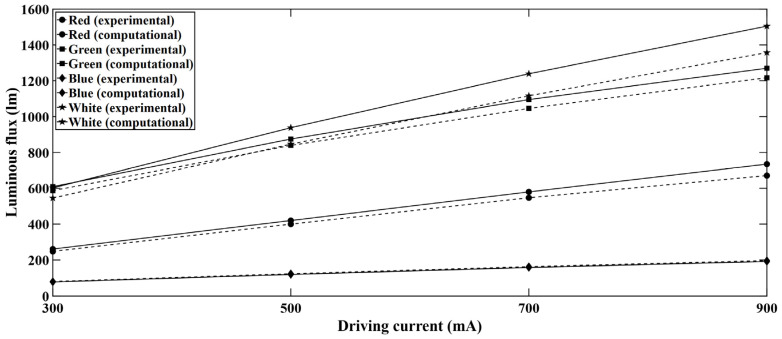
Comparison of experimental (dashed line) and computational (solid line) data for RGB-W LEDs over the light engine.

The experimental data were validated with light engine computational models. An idealized LED light engine model was developed and simulated in the ANSYS Speos package [23] for the optical study. The ray files were obtained from the LED manufacturer, and simulation parameters were used from the manufacturer’s published datasheet. Total luminous flux was observed for each color LED series and the values were compared with the experimental data for different current levels. To carry out the computation and obtain the luminous flux, an intensity sensor was defined in the simulation setup. An intensity sensor in ANSYS Speos (version 2024 R1) works as an integrating sphere. Figure 5 shows an intensity sensor along with a simplified optical model for the studied light engine. The intensity sensor was defined as a colorimetric type and for defining the X range, Y range, and wavelength; sampling was kept at 1000. The X range was –180° to 180° while the Y range was –90° to 90°. The considered wavelength for the intensity sensor was 400 nm to 700 nm. For the PCB and insulation layer of the light engine, opaque (solid body) condition was considered as the volume property while matt white condition was considered as the surface property. Ray tracing simulation was carried out using about 50 million rays. The ambient material was air in the optical simulation. A comparison of experimental and computational data is presented in Figure 6. It is noticeable that there is good agreement between the computational and experimental data. It is expected that optical power linearly increased with the increasing driving current for all LEDs.

The maximum deviation is approximately 9.8% which occurs at high driving currents. At high driving currents, the chip junction temperature increases, and the thermal dissipation becomes complex which eventually results in degraded optical performance. From Figure 6, it is observed that the computational results show a slightly higher luminous flux compared to the experimental results. The reason for this difference can be attributed to the simplification of the optical model in the computational analysis. In a practical case, the light engine set up consists of a PCB, thermal interface material, and a heat sink. These components have a wide variety of thermal and material properties. For instance, PCB itself is made of several materials and for this reason, the actual thermal behavior is difficult to comprehend. The synergistic effect of the complex resistance network across the PCB and heat sink induces additional heat generation which affects the optical performance of the LEDs.

## 4. Results and Discussions

In this study, an RGB-W LED light engine was developed for agricultural applications, and each color LED series could be driven separately. Optical and thermal performance parameters were observed simultaneously for each LED series operating separately. In this section, several optothermal performance indicators are presented and discussed from the light engine experiments.

### 4.1. Total Luminous Flux Variation

**Figure 7:**
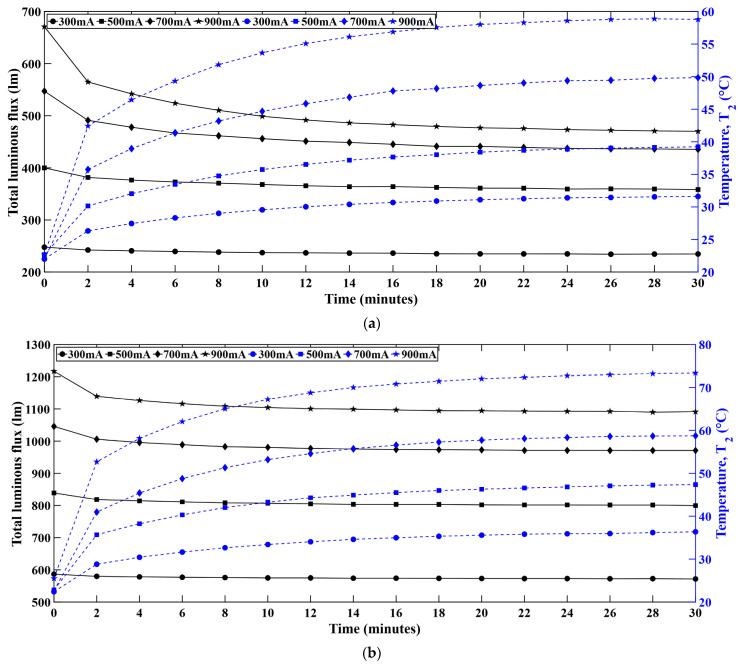

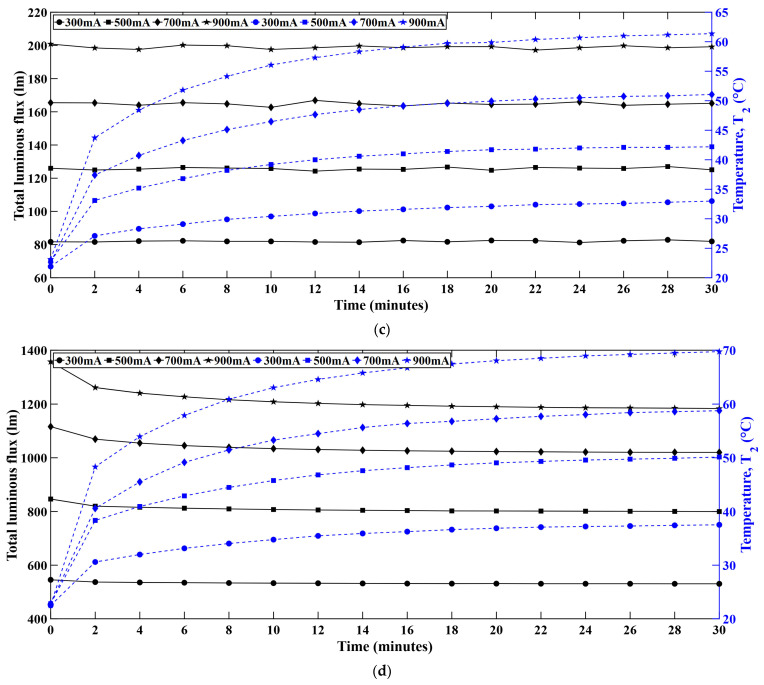
Real-time luminous flux (solid line) variation with temperature (dashed line) in (**a**) red, (**b**) green, (**c**) blue, (**d**) white LEDs.

Figure 7 represents the variation of luminous flux with time as the junction temperature keeps rising. Each LED string was driven at four current levels, i.e., 300 mA, 500 mA, 700 mA, and 900 mA. The results show that the luminous flux decreases with time as the temperature rises. Each LED series was driven for 30 min at constant current level and optical and thermal data were recorded simultaneously at 2-min intervals. The observed trend was similar for all color LED series. While the luminous flux increased as the driving current increased, the temperature continued to rise. Over the operational timespan of 30 min, it was observed that the total luminous flux dropped with the passage of time, and it was consistent in all LED strings. The thermal performance deteriorated due to the elevated junction temperature over time resulting in reduced optical performance. Apart from that, the phosphor efficiency dropped at elevated temperatures which also contributed to the optical degradation in the W-LEDs [24,25,26].

### 4.2. Variation of Luminous Efficacy

**Figure 8:**
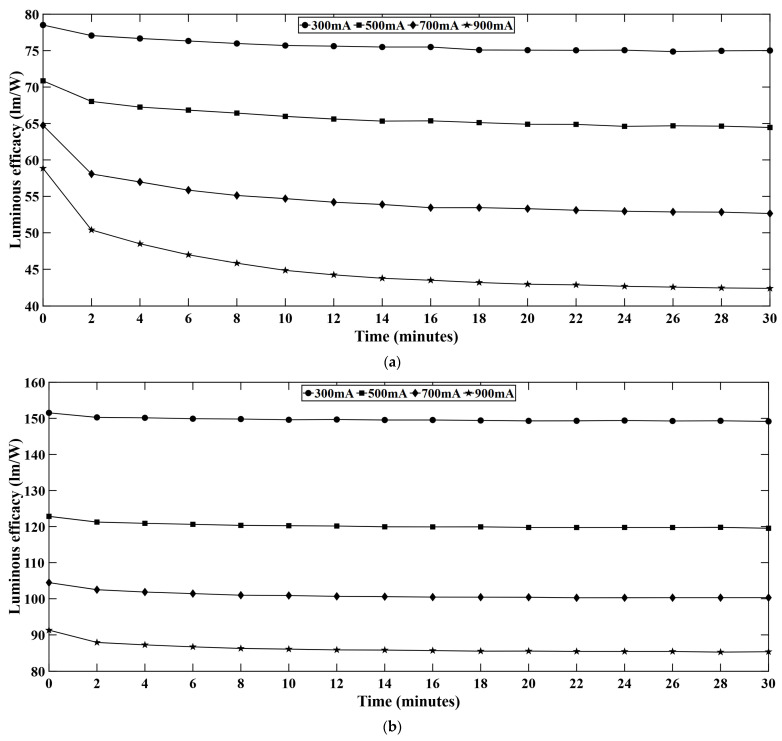

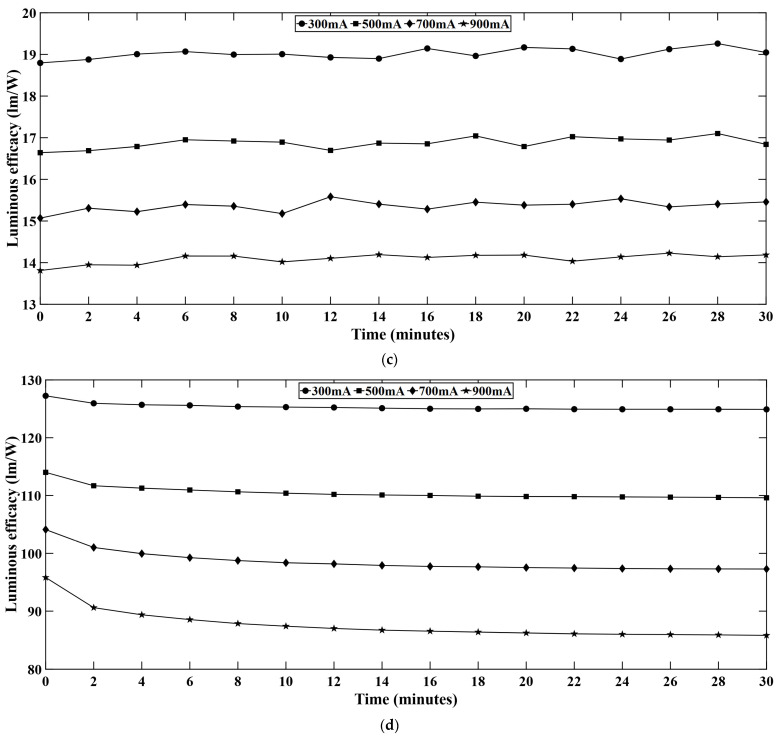
Time dependent variation of luminous efficacy in (**a**) red, (**b**) green, (**c**) blue, and (**d**) white LEDs.

Figure 8 shows the time dependent variation of luminous efficacy for each LED series. Luminous efficacy also shows variation across different current levels. It is observed that at lower current levels, the luminous efficacy is comparatively high. But with the increasing forward current, the luminous efficacy drops significantly and results in increased thermal heat generation. When LEDs are driven at higher current level, the electrical input power rises rapidly, and it may lead to net decrease in efficacy. Moreover, at higher current level, the junction temperature increases, and the internal quantum efficiency (IQE) of the system decreases. The temporal variation is also obvious due to the increased temperature in the LED system. The radiative efficiency decreases with higher driving current and with time which eventually results in reduced luminous efficacy, i.e., lumens per watt (LPW). From the experiments, it was observed that the G-LEDs provide the highest luminous efficacy followed by the W-LEDs, R-LEDs, and B-LEDs. Luminous efficacy depends on the wavelength of the emitted light and human eye sensitivity. According to the photopic sensitivity curve [27], the peak occurs at 555 nm which falls in the range of the green spectrum, while any monochromatic source emitting at 555 nm can produce maximum 683 lm/W of light output [28]. For the other monochromatic wavelengths, luminous efficacy is reduced by a factor according to photopic function [29]. For the B-LEDs, luminous efficacy does not drop as for the other LEDs. For the current study, the luminous efficacy slightly increases or remains stable over time. The reason behind this is the short operational time. While other LEDs instantly drop efficacy, B-LEDs show stability in the initial period because B-LEDs are InGaN based, and InGaN based B-LEDs show reduced electron leakage at elevated temperatures [30]. Moreover, B-LEDs show less severe efficiency droop at higher temperatures compared to G-LEDs. An efficiency droop of 47% was observed for InGaN based G-LEDs while an efficiency droop for InGaN based B-LEDs was only 18% [31]. For these reasons, B-LEDs can show increased luminous efficacy at shorter operation, i.e., during the warm-up period.

### 4.3. Correlated Color Temperature (CCT) Variation in White LED Series

**Figure 9:**
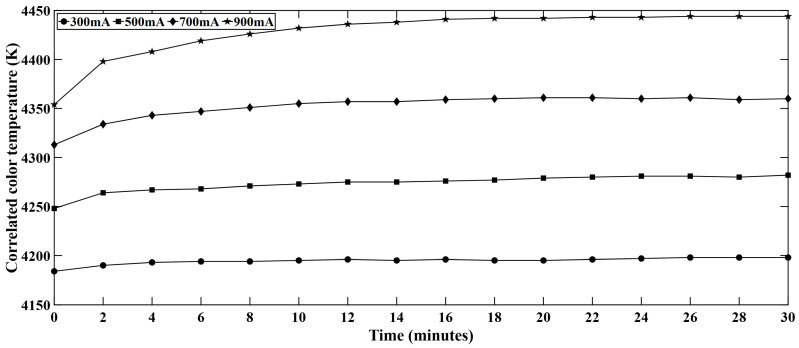
Variation of CCT with time in white LEDs.

In this study, CCT was observed along with other optical performance parameters. While the other LED colors did not show any significant variation in CCT, white LEDs showed some temporal variation in CCT. Figure 9 represents the CCT variation for different forward current levels in the white LED series. It is observed that, with the increasing forward current, the CCT increases and the CCT also increases with time. When the LED system is driven for a long period, the phosphor conversion efficiency decreases due to thermal and aging effects. This fact is also true when the LED is driven at higher current. The lower phosphor conversion efficiency tends to keep the light bluer and this phenomenon eventually results in higher CCT. Moreover, at higher currents, more electrons are injected into the LED active region which leads to higher radiative recombination rate, boosting the LED light output.

### 4.4. CRI Variation in White LED Series

**Figure 10:**
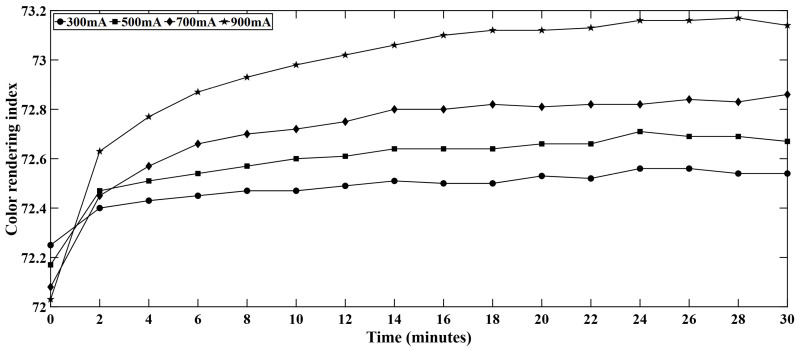
CRI variation with time in white LEDs.

Color rendering index (CRI) showed some variation with time for the white LED series. This variation in CRI is presented in Figure 10.

Since the light engine was driven for only 30 min, the improvement of the CRI can be attributed to the initial thermal stabilization of the system. Although the LEDs were driven between 300 mA to 900 mA, the LEDs used in the light engine can hold up to 1.75 A. For the safe operation of the LEDs, the maximum current level was kept at 900 mA. Since the LEDs were run at comparatively low current and for a short period, the thermal degradation and aging effect could not dominate the CRI behavior in this experiment. Another reason behind this slight CRI improvement can be attributed to the blue emission shift with time. While the junction temperature rises with time, the blue peak intensity decreases as well as the converted light shifts [32]. These shifts in both short and long wavelength regime make the spectral distribution smoother to some extent. Hence, although the luminous flux decreases with time, this spectral adjustment enhances the overall color quality, leading to a small but consistent rise in CRI.

### 4.5. Input Electrical Power

**Figure 11:**
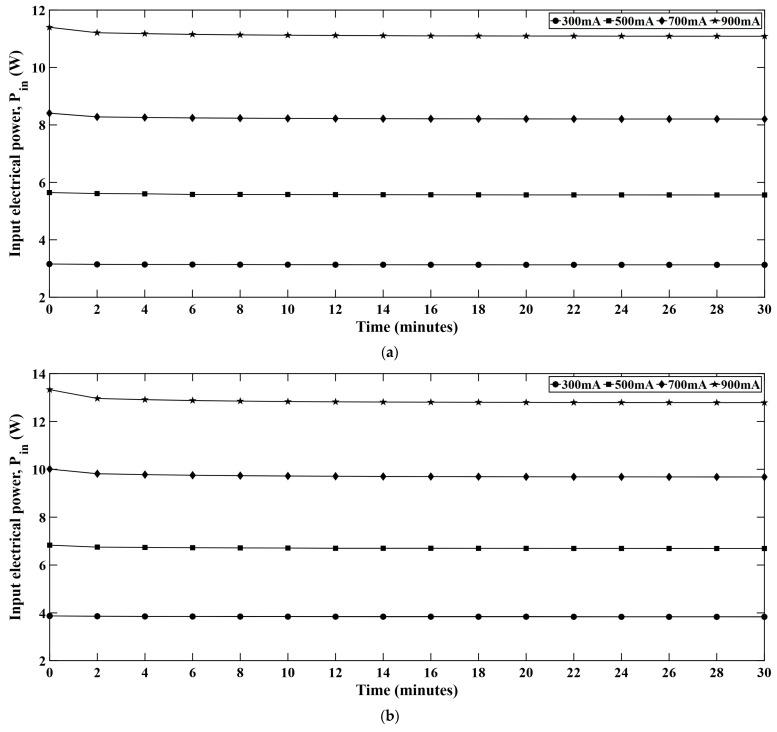

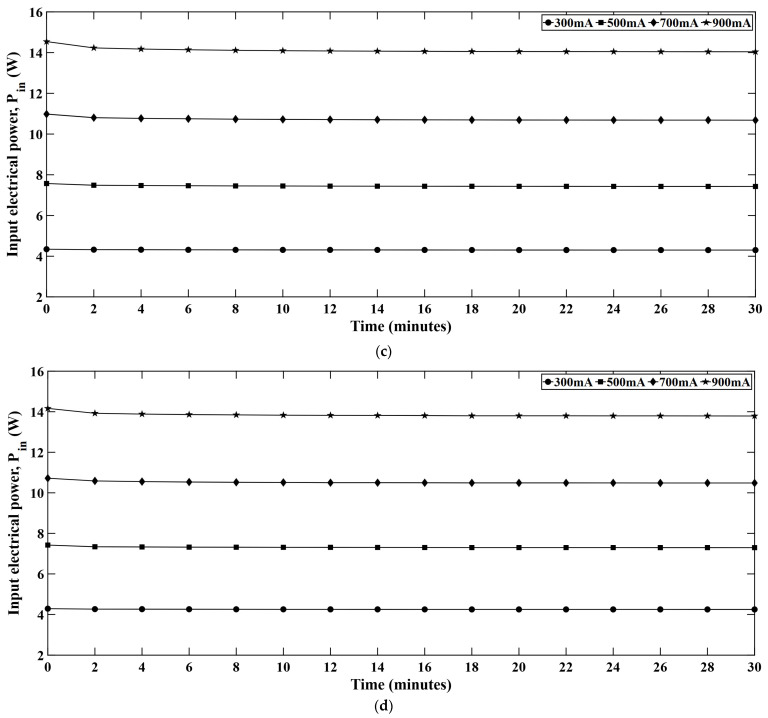
Time dependent variation of input electrical power in (**a**) red, (**b**) green, (**c**) blue, and (**d**) white LEDs.

Figure 11 represents time dependent electrical power input for different LED strings. The input powers are shown for the red, green, blue, and white LED series and it was observed that the input electrical power drops as the light engine keeps operating. This is because, as the LEDs warm up, the forward voltage decreases. Since the LEDs are driven at constant current, the reduced forward voltage results in a lower input power. At the beginning of the test, LEDs remain cooler and draw high electrical power from the input source. However, as the operating time increases, the junction temperature becomes high and the forward voltage of the LEDs starts to drop. This fact leads to the lower input power in LED systems when driven for longer time periods.

### 4.6. Radiant Flux

**Figure 12:**
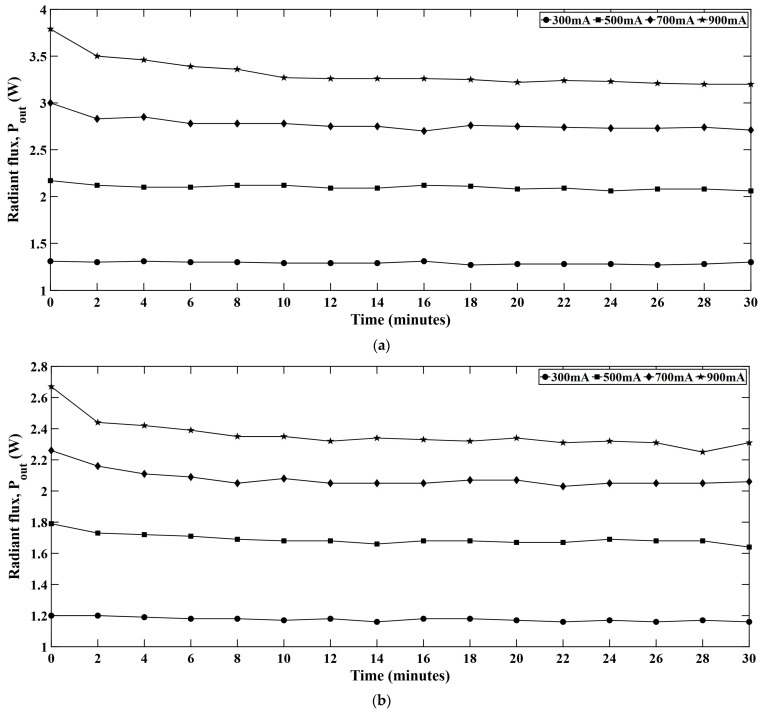

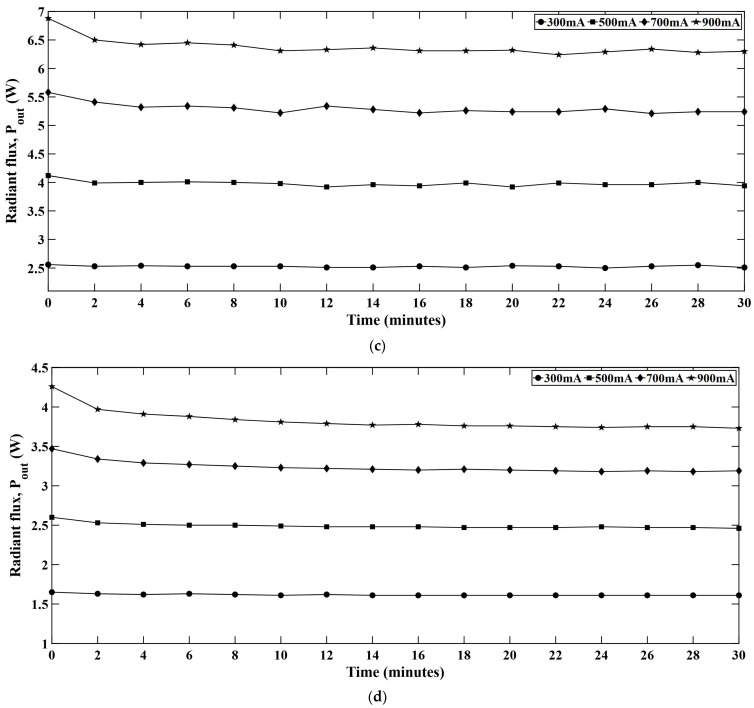
Time dependent radiant flux at different current levels for (**a**) red, (**b**) green, (**c**) blue, and (**d**) white LEDs.

Figure 12 presents the radiant flux with time at different constant current levels. From different LED strings, it is observed that the blue string shows the highest radiant flux among all the colors. B-LEDs are typically based on an InGaN semiconductor structure and possess higher IQE compared to the green and red counterparts [33]. The InGaN/GaN based B-LEDs can reach an electrical to optical power conversion efficiency of more than 80% which also confirms the high IQE of B-LEDs [34]. For this reason, B-LEDs show higher photon generation per unit electrical power input. Moreover, due to the shorter wavelength, B-LEDs show less scattering or absorption behavior within the encapsulant or optical components which allows more light to escape the LED package.

### 4.7. Chip Heat Generation Rate

**Figure 13:**
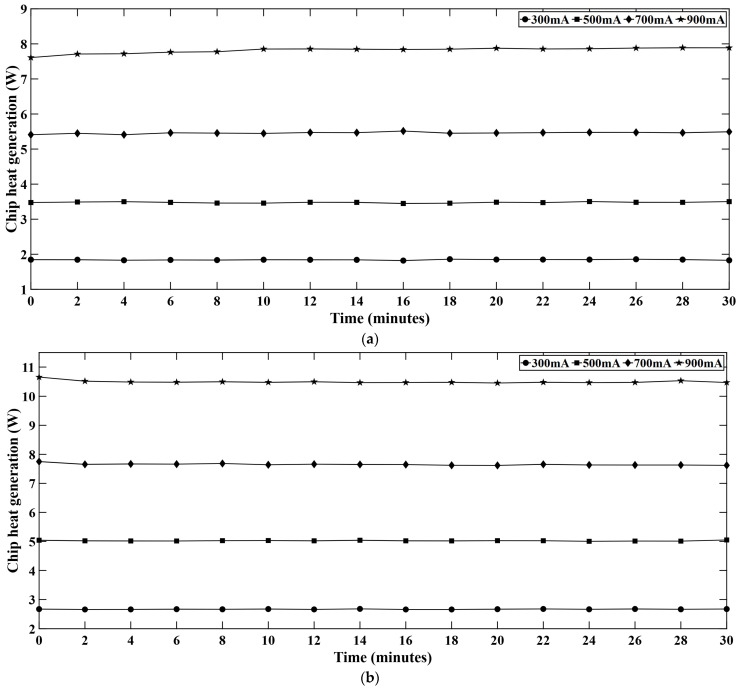

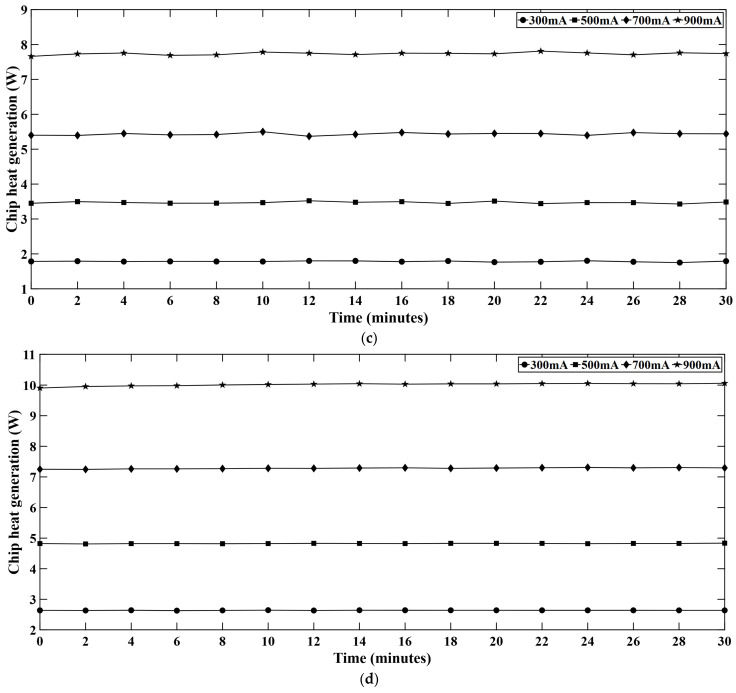
Chip heat generation rate at different current levels in (**a**) red, (**b**) green, (**c**) blue, and (**d**) white LEDs.

Figure 13 represents the chip heat generation rate in different LED series. The heat generation rate remains slightly off when the light engine is turned on. With the passage of time, the heat generation rate keeps rising and becomes steady at a certain point. Among the different color LED strings, the green series shows the highest heat generation rate followed by the white series. As we discussed in Section 4.6, the B-LEDs are the most efficient in converting electrical power into photons; most of the power is converted as radiant flux. That is why the chip heat generation rate is comparatively low in the blue series. On the contrary, G-LEDs have low internal efficiency [35,36] and most of the electrical power is converted into non-radiative heat resulting in a very high heat generation rate in the chip.

### 4.8. Temperature Variation at the Light Engine PCB

**Figure 14:**
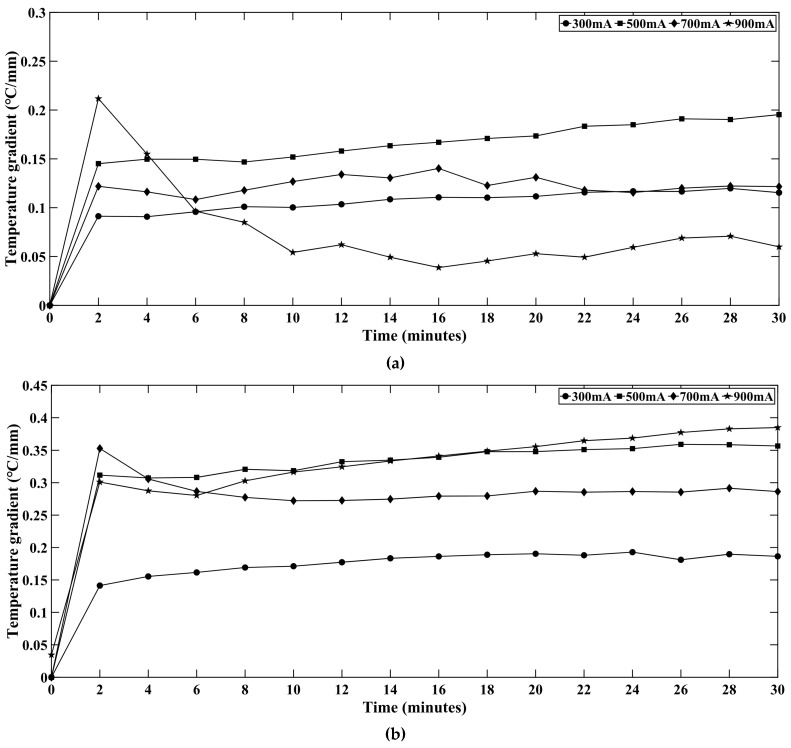

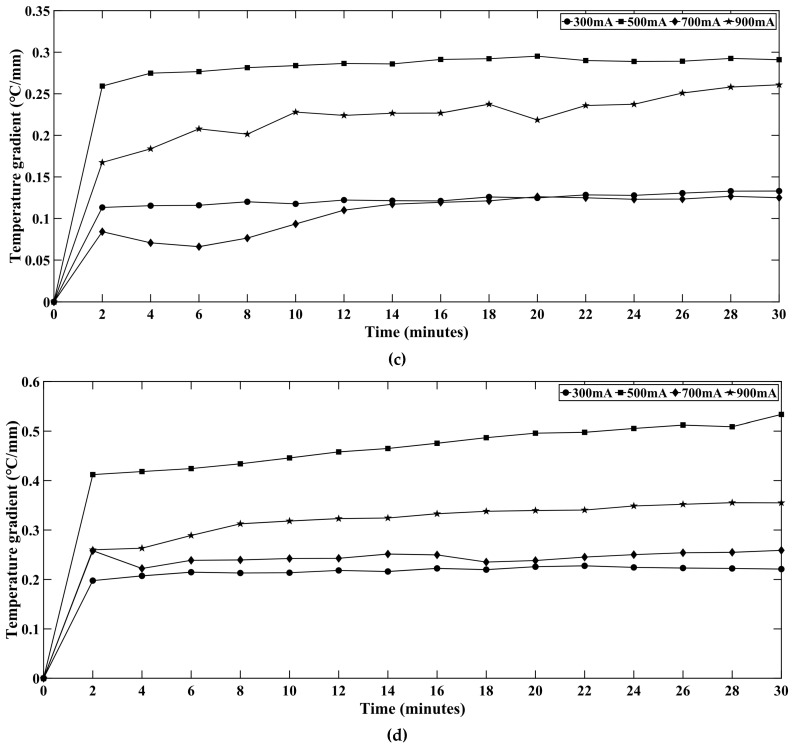
Temperature gradients between the center and midway down the PCB bottom for (**a**) red, (**b**) green, (**c**) blue, and (**d**) white LEDs.

To observe the thermal performance across the light engine, two thermocouples were inserted at the backside of the aluminum PCB. One TC measured the temperature at the center (T_2_) and another one measured the temperature midway down the PCB bottom (T_1_). Table 1 presents both the temperatures for the red, green, blue, white LED series. The current LED system consists of an LED light engine on an aluminum PCB, with thermal tape and a heat sink underneath it. The maximum temperature of such systems is reached at the junction of the chip followed by the PCB. Hence, the most practical and closest point to measure temperature is the backside of the PCB. It is observed that the temperature T_1,_ i.e., the temperatures 10 mm away from the PCB backside center and temperature T_2_ (center of PCB backside) show consistent behavior. The thermal gradients between these two points are shown in Figure 14 for all LED strings. The temperature gradient is positive across all cases, i.e., temperature T_2_ is greater than T_1_. Initially the temperature gradient remains quite high because there is a significant difference between the two points’ temperatures. However, the system reaches steady state gradually and the temperature gradient keeps falling or becomes stable. The reason is the thermal spreading across the system. Initially the system does not get sufficient time to reach a steady state but after operating for 25–30 min, the system reaches a steady-state, and the temperature gradient also becomes stable.

### 4.9. Spectral Flux Variation with Time at Different Current Levels

**Figure 15:**
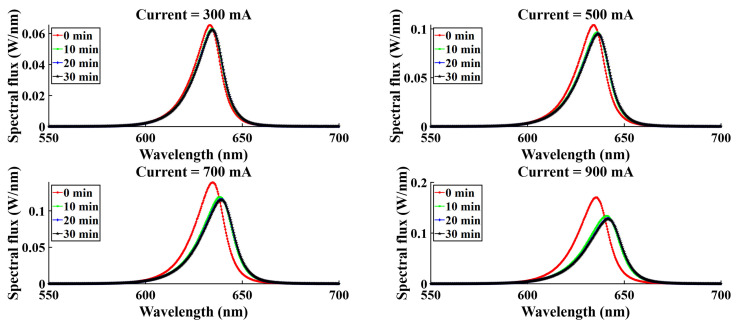
Spectral variation with time for R-LEDs.

**Figure 16:**
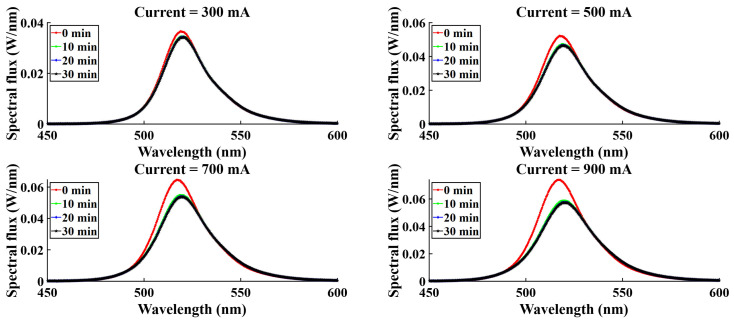
Spectral variation with time for G-LEDs.

**Figure 17:**
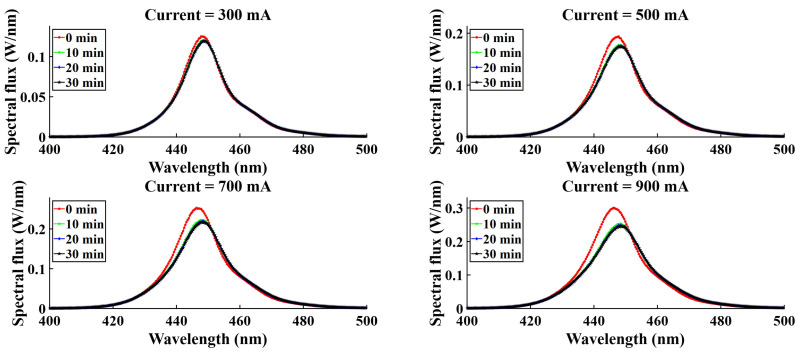
Spectral variation with time for B-LEDs.

**Figure 18:**
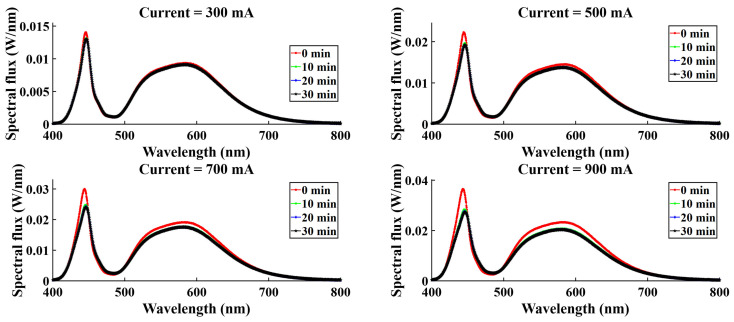
Spectral variation with time for W-LEDs.

For four different current levels (300 mA, 500 mA, 700 mA, 900 mA), spectral variation was observed, and spectral flux showed variation with time. Figure 15, Figure 16, Figure 17, and Figure 18 represent the temporal variation of the spectral flux for the red, green, blue, and white LEDs, respectively. The spectral variation is obvious across different current densities due to the variation in chip heat generation. However, spectral flux varies with time also. With time, the chip junction temperature rises, and optical performance degrades simultaneously. In addition to the temperature rising, the phosphor degradation and aging effect also play a critical role in the spectral shift as well as the peak intensity drop.

### 4.10. Experimental Uncertainty Analysis

In the experimental measurements, uncertainties are inevitable. In this study, major sources of uncertainties originated from the instruments used. In order to calculate the uncertainties of the derived parameters, we need to use the governing formula containing the measured parameters. For instance, the input power equation is P=VI. The maximum uncertainty in input power is calculated by using Equation (1):

(1) $$\frac{u_{P}}{P}=\sqrt{{\left(\frac{u_{V}}{V}\right)}^{2}+{\left(\frac{u_{I}}{I}\right)}^{2}}$$

Since the maximum power of the DC power supply is 1200 W, different current–voltage combinations (e.g., 300 V-4 A, 600 V-2 A, etc.) can be used to find out the power uncertainty. The maximum power uncertainty is 4.02 W which is reported in Table 2. For the optical parameters, the uncertainties were obtained from the calibration certificate received from the integrating sphere manufacturer. The experimental uncertainties of different measured and derived parameters are presented in Table 2.

## 5. Conclusions

The current study was conducted to observe several optothermal behaviors of an in-house developed and manufactured RGB-W LED light engine for agricultural applications. Temporal behavior changes in the relevant performance parameters were also observed. The following conclusions can be made from this study:

(a) Luminous flux decreases with time at any current level, and this is due to thermal degradation of the package while in operation. However, the B-LED series provides an almost constant luminous flux level throughout the operation time. That is because the B-LEDs are most efficient in converting electrical energy into photons and generating less heat. Also, having an operational time of only 30 min, there is no significant flux drop in the B-LEDs. However, in case of longer operation, B-LEDs also show a drop in luminous flux.
(b) The luminous efficacy keeps falling as the driving current increases. Additionally, there are some temporal variations in the luminous efficacy. At higher current levels, the junction temperature increases, and the internal quantum efficiency reduces which results in low luminous efficacy. However, B-LEDs do not show any efficacy drop during the operational time. In fact, they showed some enhancement because of the high conversion efficiency and less current leakage of the InGaN based B-LEDs. Moreover, the efficiency droop is very low for B-LEDs which also contributes to the consistent luminous efficacy over a short operational time.
(c) With time, the input electrical power decreases because the LEDs are driven at constant current and the forward voltage keeps falling when the system is in operation. This is because the LED chips heat up once turned on and the forward voltage drops compared to the initial cool condition.
(d) B-LEDs show the maximum radiant flux because of having the highest conversion efficiency. On the other hand, G-LEDs produce the most heat as they show the least conversion efficiency. The efficiency droop for G-LEDs is around 47%, while B-LEDs show only 18% efficiency droop.
(e) The temperature gradient at the PCB backside shows that temperature T_2_ is greater than T_1_. That is because thermocouple 2 is directly under the central chip while the other thermocouple is 10 mm away from the center. The temperature gradient remains high during the initial operation time and gradually reaches the steady state condition because of thermal spreading.
(f) Spectral flux shows temporal variation for all color LEDs at any current level. Junction temperature rise, carrier leakage, phosphor degradation, etc. can be attributed to the potential causes of this spectral shift. For these reasons, the peak wavelength drops too.

In this study, the light engine was operated for 30 min, but in practical applications, it needs to operate continuously. Continuous and reliable operation can ensure proper welfare and productivity in any agricultural application. Hence, it is recommended to extend the testing duration so that the optical parameters such as radiant flux, spectral flux, luminous efficacy, etc., and their degradation trend can be captured effectively. In addition to that, applying a lifetime prediction model, such as the Arrhenius equation, needs to be incorporated. This can provide quantitative estimates of the LED lifetime under different operating temperatures and stress conditions. This approach will enable a more reliable assessment of long-term performance, creating a benchmark for design improvement and operational strategy for enhanced reliability.

## Figures and Tables

**Table 1:** Temperature measurements for multiple LED series.

Color	Current (mA)	300		500		700		900	
	Time (min.)	T_1_ (°C)	T_2_ (°C)	T_1_ (°C)	T_2_ (°C)	T_1_ (°C)	T_2_ (°C)	T_1_ (°C)	T_2_ (°C)
Red	0 2 4 6 8 10 12 14 16 18 20 22 24 26 28 30	22.1 25.4 26.6 27.4 28.0 28.6 29.0 29.3 29.6 29.8 30.0 30.1 30.2 30.3 30.4 30.5	22.0 26.3 27.5 28.3 29.0 29.6 30.0 30.4 30.7 30.9 31.1 31.3 31.4 31.5 31.6 31.6	22.8 28.7 30.5 32.0 33.3 34.2 34.9 35.5 36.0 36.3 36.7 36.9 37.0 37.1 37.2 37.3	22.7 30.2 32.0 33.5 34.8 35.7 36.5 37.2 37.7 38.0 38.4 38.7 38.9 39.0 39.1 39.2	22.1 35.2 38.2 40.5 42.4 44.0 45.1 46.1 46.9 47.5 48.0 48.4 48.7 48.8 49.1 49.2	22.0 35.7 38.9 41.4 43.2 44.7 45.9 46.8 47.8 48.2 48.6 49.0 49.4 49.5 49.7 49.8	22.8 40.3 44.9 48.4 51.0 53.1 54.5 55.6 56.5 57.1 57.5 57.8 58.0 58.1 58.2 58.2	22.7 42.4 46.4 49.3 51.8 53.7 55.1 56.1 56.9 57.6 58.0 58.3 58.6 58.8 58.9 58.8
Green	0 2 4 6 8 10 12 14 16 18 20 22 24 26 28 30	22.5 27.4 28.9 30.0 31.0 31.7 32.3 32.8 33.1 33.4 33.7 33.9 34.0 34.1 34.3 34.5	22.4 28.8 30.4 31.6 32.6 33.4 34.0 34.6 35.0 35.3 35.6 35.8 35.9 36.0 36.2 36.4	22.9 32.6 35.2 37.2 38.8 40.1 41.0 41.6 42.1 42.5 42.8 43.1 43.3 43.5 43.7 43.8	22.8 35.7 38.3 40.3 42.0 43.3 44.3 44.9 45.5 46.0 46.3 46.6 46.8 47.1 47.2 47.4	22.8 37.4 42.4 45.9 48.6 50.5 51.9 53.0 53.8 54.5 54.9 55.3 55.5 55.8 55.8 55.9	22.6 41.0 45.4 48.8 51.3 53.2 54.6 55.8 56.6 57.3 57.8 58.1 58.3 58.6 58.7 58.8	23.1 47.7 54.1 58.0 61.7 64.1 65.6 66.7 67.4 68.1 68.3 68.6 68.8 69.1 69.2 69.3	23.0 58.7 66.6 69.9 68.1 69.4 70.9 72.0 72.8 73.4 73.8 74.2 74.5 74.7 74.8 74.9
Blue	0 2 4 6 8 10 12 14 16 18 20 22 24 26 28 30	22.0 26.0 27.1 28.0 28.7 29.3 29.7 30.0 30.4 30.7 30.9 31.1 31.2 31.3 31.5 31.6	21.9 27.1 28.3 29.1 29.9 30.4 30.9 31.3 31.6 31.9 32.1 32.4 32.5 32.6 32.8 33.0	22.7 30.5 32.5 34.1 35.4 36.3 37.1 37.7 38.1 38.5 38.7 38.9 39.1 39.2 39.2 39.3	22.8 33.1 35.2 36.8 38.2 39.2 40.0 40.6 41.0 41.4 41.7 41.8 42.0 42.1 42.1 42.2	22.7 36.6 40.0 42.6 44.4 45.5 46.6 47.3 47.9 48.4 48.7 49.0 49.3 49.5 49.6 49.8	22.6 37.4 40.7 43.3 45.1 46.5 47.7 48.5 49.1 49.6 49.9 50.3 50.5 50.7 50.8 51.1	23.3 42.1 46.6 49.7 52.1 53.8 55.1 56.1 56.8 57.4 57.7 58.0 58.3 58.5 58.6 58.8	23.1 43.7 48.4 51.8 54.1 56.1 57.3 58.3 59.1 59.7 59.9 60.4 60.7 61.0 61.2 61.4
White	0 2 4 6 8 10 12 14 16 18 20 22 24 26 28 30	22.6 28.6 29.9 31.0 31.9 32.6 33.3 33.7 34.0 34.4 34.6 34.8 35.0 35.1 35.2 35.3	22.5 30.6 32.0 33.1 34.0 34.8 35.5 35.9 36.2 36.6 36.9 37.1 37.2 37.3 37.4 37.5	22.9 34.2 36.7 38.7 40.1 41.3 42.2 42.9 43.4 43.8 44.1 44.4 44.5 44.6 44.8 44.8	22.8 38.3 40.9 42.9 44.5 45.8 46.8 47.6 48.2 48.7 49.1 49.3 49.6 49.7 49.9 50.1	22.9 38.0 43.3 46.8 49.1 50.9 52.1 53.1 53.9 54.4 54.9 55.3 55.5 55.9 56.0 56.2	22.8 40.6 45.5 49.2 51.5 53.3 54.5 55.6 56.4 56.8 57.3 57.7 58.0 58.4 58.6 58.8	22.6 45.7 51.3 55.0 57.8 59.9 61.4 62.6 63.4 64.1 64.7 65.1 65.5 65.7 65.9 66.2	22.5 48.3 54.0 57.9 60.9 63.1 64.6 65.8 66.8 67.5 68.0 68.5 68.9 69.2 69.5 69.7

**Table 2:** Experimental uncertainties.

Parameter	Parameter Type	Device	Model	Range	Accuracy	Maximum Uncertainty
Temperature (°C)	Measured	Thermocouple (T-type)	Omega 5TC-TT-T-30–36-ROHS	0–150	±0.75%	1
		DAQ	Agilent 34970A	−100 to 400	±1	4%
Voltage (V)	Measured	Power supply	Chroma 62012P-600-8	0–600	0.05% + 0.05%F.S.	0.6
Current (A)	Measured	Power supply	Chroma 62012P-600-8	0–8	0.1% + 0.1%F.S.	0.016
Input power (W)	Derived	Power supply	Chroma 62012P-600-8	0–1200	-	4.02
Luminous flux (%)	Measured	Integrating sphere	Labsphere illumia plus 40″ base sphere	-	-	1
CCT (K)	Measured	Integrating sphere	Labsphere illumia plus 40″ base sphere	-		±7

## Data Availability

Data are contained within the article.

## Abbreviations

The following abbreviations are used in this manuscript:

CEA	Controlled environment agriculture
CCT	Correlated color temperature
CRI	Color rendering index
IoT	Internet of things
IQE	Internal quantum efficiency
LED	Light emitting diode
LPW	Efficacy (lumen/watt)
PCB	Printed circuit board
RGB-W	Red, green, blue, white
TC	Thermocouple
